# Photobiomodulation may enhance cognitive efficiency in older adults: a functional near-infrared spectroscopy study

**DOI:** 10.3389/fnagi.2023.1096361

**Published:** 2023-07-20

**Authors:** Tsz-lok Lee, Agnes S. Chan

**Affiliations:** ^1^Neuropsychology Laboratory, Department of Psychology, The Chinese University of Hong Kong, Shatin, Hong Kong SAR, China; ^2^Research Centre for Neuropsychological Well-Being, The Chinese University of Hong Kong, Shatin, Hong Kong SAR, China

**Keywords:** brain stimulation, memory, cognitive efficiency, mental effort, photobiomodulation

## Abstract

**Introduction:**

The relative oxygenated hemoglobin (HbO) measured using functional near-infrared spectroscopy (fNIRS) has been considered as an index for cognitive loading, with the more difficult the task, the higher the level. A previous study reported that young adults who received transcranial photobiomodulation (tPBM) showed a reduced HbO of a difficult task, suggesting that tPBM may enhance cognitive efficiency. The present study further investigated the effect of tPBM on cognitive efficiency in older adults.

**Methods:**

Thirty participants received a single tPBM on the forehead for 350 s. Before and after tPBM, their HbO in the visual span task with various difficulties was measured with fNIRS.

**Results:**

After tPBM, participants exhibited significantly lower HbO in a harder (span 7) but not an easier level (span 2) of the task, but their behavioral performance remained unchanged. In addition, factors affecting the reduction of HbO were examined, and the results showed that individuals with better memory (as measured by a 30-min delayed recall test) showed more reduction of HbO.

**Discussion:**

The results suggest that tPBM may enhance cognitive efficiency, with individuals with better memory tend to benefit more.

## 1. Introduction

Photobiomodulation (PBM) is a non-invasive technique that delivers red to near-infrared light, with wavelengths ranging from 600 to 1,100 nm, to targeted sites of the body (Hamblin et al., 2018). When the light is shone on the scalp and impacts the brain through the skull, this procedure is referred to as transcranial PBM (tPBM). Although most of the light is absorbed or scattered by non-neural tissue as it penetrates through the scalp, skull, and cerebrospinal fluid, a small fraction of the delivered light may reach neural tissue (Henderson and Morries, 2015; Salehpour et al., 2019a), because biological tissue is relatively transparent to red to near-infrared light (Bashkatov et al., 2005). The mechanisms underlying the action of PBM are multifaceted and involve intracellular activities, extracellular adaptations, and morphological alterations. Previous studies reported positive effects when tPBM was applied to patients with traumatic brain injury (Naeser et al., 2014; Longo et al., 2020), stroke (Lampl et al., 2007; Zivin et al., 2009), mild cognitive impairment (MCI) (Chan et al., 2021b,c), dementia (Saltmarche et al., 2017; Chao, 2019), and mood disorders (Schiffer et al., 2009; Cassano et al., 2015; Kerppers et al., 2020).

The effect of tPBM on cognitive function was also observed in a single tPBM session. Blanco et al. (2015) recruited 30 young healthy adults, and half of them received an 8-min tPBM on the right prefrontal cortex, whereas another half received sham stimulation. The results showed that participants who received the tPBM made fewer errors and showed improved set-shifting ability in a test of executive function as compared to those who received the sham tPBM. Similarly, another study on young healthy adults also showed shorter reaction time in the Go/No-Go task after a 2.5-min tPBM stimulation on the Fp2 region (i.e., right prefrontal cortex), which suggested improved sustained attention (Jahan et al., 2019). The beneficial effect of tPBM was not restricted to young adults; it was also found that cognitive function was enhanced in healthy older adults. A study found that healthy older adults had significant improvement in action selection, inhibitory ability, and mental flexibility after receiving a 7.5-min real tPBM, whereas those who received a sham stimulation did not show any significant change (Chan et al., 2019a).

In addition to the improvement in cognitive function, we explored the change in brain hemodynamic response underlying this improvement using functional near-infrared spectroscopy (fNIRS) previously. fNIRS is a non-invasive functional neuroimaging technique that uses near-infrared light to estimate the changes in cortical concentrations of oxygenated hemoglobin (HbO) and deoxygenated hemoglobin (HbR) in response to neural activity. It was found that fNIRS is sensitive to variations in mental effort, especially in the prefrontal cortex, in completing a cognitive task. Specifically, increased concentration of HbO and decreased concentration of HbR in the prefrontal cortex were observed when a task became more complex (Vassena et al., 2019; Csipo et al., 2021) and when a difficult task was anticipated (Vassena et al., 2019). Previous fNIRS studies found that tPBM may enhance cognitive efficiency by reducing the cognitive efforts needed to complete a task that requires high memory loads (Chan et al., 2021a,b). In specific, in a study, 33 healthy young adults were divided into either the experimental group or the control group (Chan et al., 2021b). The experimental group received a 350-s active tPBM on the prefrontal cortex, whereas the control group received sham stimulation. In this experiment, participants were required to complete a working memory task (i.e., *n*-back task) with easy (i.e., *0*-back) and difficult (i.e., *3*-back) conditions. Their hemodynamic responses during the task before and after the tPBM session were measured using fNIRS. The results showed that the experimental group exhibited a significantly reduced working memory-related HbO (i.e., HbO in *3*-back minus HbO in *0*-back) in the prefrontal cortex after tPBM, but this change was not observed in the control group. In addition, after tPBM, both groups showed similar *n*-back performance in both *0*- and *3*-back conditions as compared to their baseline. Combining the fNIRS and behavioral results, it was concluded that the experimental group could achieve a similar level of performance with less cognitive effort, indicated by reduced working memory-related HbO. This suggests that tPBM enhances the cognitive efficiency of individuals.

In addition, an improvement in cognitive efficiency was also observed in older adults with MCI (Chan et al., 2021a). In this study, nine MCI participants received a single 350-s real tPBM session on the prefrontal cortex, whereas another nine MCI participants received sham stimulation. All the participants were asked to complete a visual span task with fNIRS before and after tPBM. The results showed that only participants who received a real tPBM, but not those who received the sham stimulation, had significantly improved task performance and also reduced HbO during the task. These findings suggested that tPBM may reduce the cognitive efforts needed to complete the cognitive task and thus improve the cognitive performance of MCI patients.

This study aimed to extend prior research by investigating the effects of a single tPBM session on enhancing cognitive efficiency in older adults. Besides, as we observed variation of effect among individuals after receiving tPBM in our center, we speculated that there might be factors that can predict the size of the effect of tPBM. The second purpose was to examine factors that may predict the effectiveness of tPBM on different individuals. Based on previous results on young adults (Chan et al., 2021a), it was anticipated that individuals with better memory ability might demonstrate more reduction of HbO after the tPBM.

## 2. Materials and methods

### 2.1. Participants

Thirty adults aged between 50 and 80 years were recruited through university mass mail and the department subject database. Participants were eligible if they could understand Chinese and had normal or corrected-to-normal vision. Participants were excluded if they reported any history of psychiatric disorder, neurological disorder, head injury, or epilepsy. All participants had a score below 1 in the Clinical Dementia Rating (Morris, 1997) and a score below 9 in the Functional Activities Questionnaire (Pfeffer et al., 1982), indicating the absence of dementia. This study was conducted in accordance with the Declaration of Helsinki of the World Medical Association Assembly and was approved by the Joint Chinese University of Hong Kong–New Territories East Cluster Clinical Research Ethics Committee. Informed consent was obtained from all participants. This study was not preregistered.

### 2.2. Measures

#### 2.2.1. Neuropsychological assessments

Participants’ memory, executive function, attention, and mental flexibility were assessed by standardized neuropsychological tests, including the Hong Kong List Learning Test (HKLLT; Chan, 2006), Rey-Osterrieth Complex Figure Test (Rey-O; Meyers and Meyers, 1995), Digit Span Test (Wechsler, 2008), and Category Fluency Test (Chan and Poon, 1999). In the HKLLT, participants were verbally presented with a list of Chinese vocabulary items three times and were required to recall as many items as possible after each trial. They were then asked to recall the list again after 10 and 30 min. The total learning score, which ranged from 0 to 48, was calculated as the sum of the number of items correctly recalled across all three learning trials. The number of items correctly recalled in the 10-min and 30-min delayed recall trials was also computed and ranged from 0 to 16. A higher score indicates better memory performance. Participants’ anxiety and depressive symptoms were measured using the ten-item short form of the Geriatric Anxiety Scale (GAS; Mueller et al., 2015) and the 15-item short form of the Geriatric Depression Scale (GDS; Sheikh and Yesavage, 1986), respectively.

#### 2.2.2. fNIRS task

**FIGURE 1:**
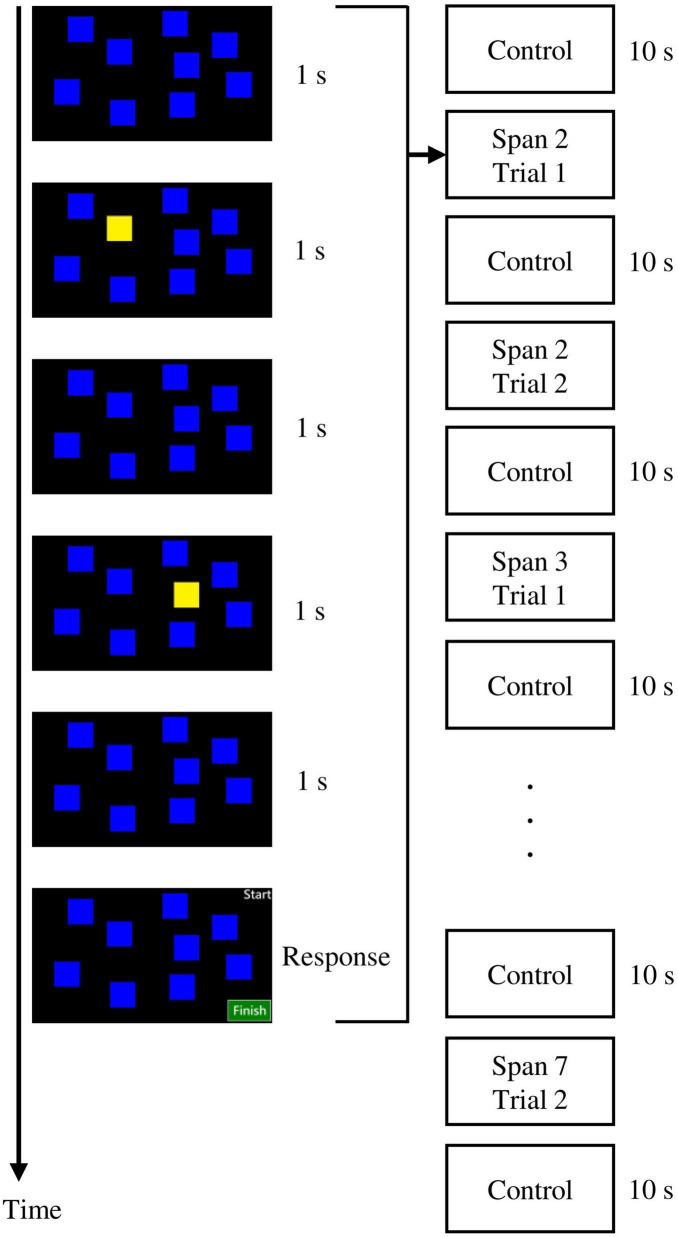
The visual span task employed in the present study.

A computerized visual span task was employed to assess the visual memory of the participants (Figure 1). The details of the task were described in a previous tPBM study (Chan et al., 2021a). In this task, each trial started with nine blue square blocks for 1 s. Next, a sequence of blue squares was turned into yellow one by one in a second each. Participants were asked to memorize and recall the sequence by clicking the square blocks using a computer mouse. The task started with a sequence of two blocks to seven blocks. There were two trials for each span. The highest correct span was scored as the dependent variable.

#### 2.2.3. Hemodynamic measures

**FIGURE 2:**
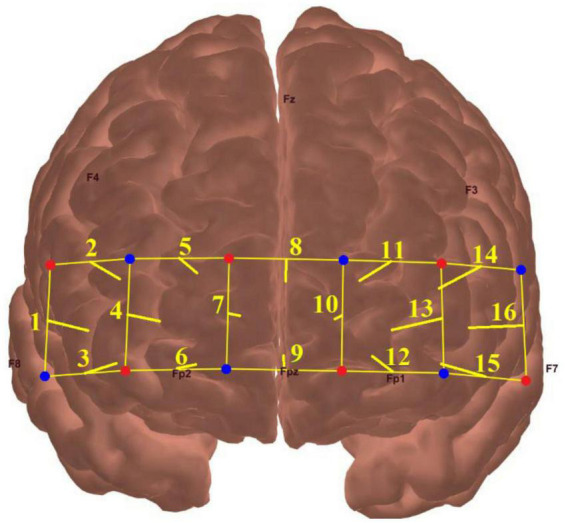
The locations of fNIRS source probes (red dots), detector probes (blue dots), and the 16 measurement channels (yellow lines).

A 16-channel fNIRS system (model OEG-SpO2, Spectratech Inc., Tokyo, Japan) was employed to measure the mental effort made by the participants during the visual span task at each difficulty level. This machine utilized near-infrared light with wavelengths of 770 and 840 mm to calculate participants’ relative HbO based on the modified Beer-Lambert law (Delpy et al., 1988). The six pairs of emitter and detector probes were arranged in a 2 rows × 6 columns matrix on the forehead of each participant, with a fixed distance of 3 cm in between (Figure 2).

### 2.3. Transcranial photobiomodulation

Transcranial photobiomodulation was applied using a custom-built device. The device contained nine clusters, and each cluster consisted of three light-emitting diodes (LEDs). Each LED emitted light of wavelength 810 nm in a continuous wave at an irradiance of 20 mW/cm^2^ and a fluence of 7 J/cm^2^. The nine clusters were placed in the prefrontal region of the participants approximately at F7, AF7, Fp1, Fpz, Fp2, AF8, F8, Fz, and Cz according to the international 10/10 system. The duration of tPBM lasted for 350 s. That is, the total power and total energy of each cluster were 60 mW and 21 J, respectively. All the participants did not report any uncomfortable feelings or side effects during and after tPBM.

### 2.4. Procedures

All participants underwent neuropsychological assessments and were asked to perform the computerized visual span task with fNIRS measurement. Immediately after the pre-assessment, all participants received a single real tPBM session. The same visual span task was performed again with fNIRS in post-assessment. There was no time delay period between the assessments and the tPBM session.

### 2.5. Data preprocessing and analysis

For the fNIRS data, a 0.10 Hz low-pass filter with a slope of 60 dB/octave was applied to remove high-frequency noise during the task. Correlation-based signal improvement was then used to reduce the movement artifacts of the signals (Cui et al., 2010). Baseline correction was performed by subtracting the average HbO before the start of the visual span task, which lasted for 10 s. The corrected fNIRS signal was then averaged across spans and participants.

Analysis of variance (ANOVA) and chi-squared test were conducted to compare the between-group difference at baseline. Repeated measures ANOVA was performed to evaluate the interaction of the within-subject factors. Mixed ANOVA was performed to evaluate the interaction of between-subject × within-subject factors. Greenhouse Geisser correction was performed if the sphericity assumption was violated. *Post-hoc* paired *t*-tests were performed to investigate any significant within-group differences. The Holm-Bonferroni correction was applied to adjust the *p*-values to control for multiple comparisons. Correlation and stepwise regression analysis was performed to determine factors that predicted the change in cognitive efficiency after tPBM. The effect sizes of the tests were estimated using Cohen’s d and partial eta squared ($\eta {}_{p}^{2}$).

## 3. Results

### 3.1. Changes in relative HbO after tPBM

The relative HbO at pre-assessment was analyzed first. There was a significant span (span 2 to 7) × channel (channel 1 to 16) interaction, *F*(9.25,268.30) = 2.22, *p* = 0.020, $\eta_{p}^{2}$ = 0.071. The main effect of the span was significant, *F*(1.80,52.28) = 56.60, *p* < 0.001, $\eta_{p}^{2}$ = 0.66, in which the linear trend was the most pronounced, *F*(1,29) = 77.71, *p* < 0.001, suggested that the more difficult of the task, the higher the relative HbO exhibited. The main effect of the span was found in all 16 channels, *F* ≥ 35.80, *p* < 0.001, $\eta_{p}^{2}$ = 0.55. The *Post hoc* analysis found that the mean relative HbO was higher than its previous span (i.e., relative HbO at span*_*n*_* > span_*n*–1_, adjusted *p* ≤ 0.006).

**FIGURE 3:**
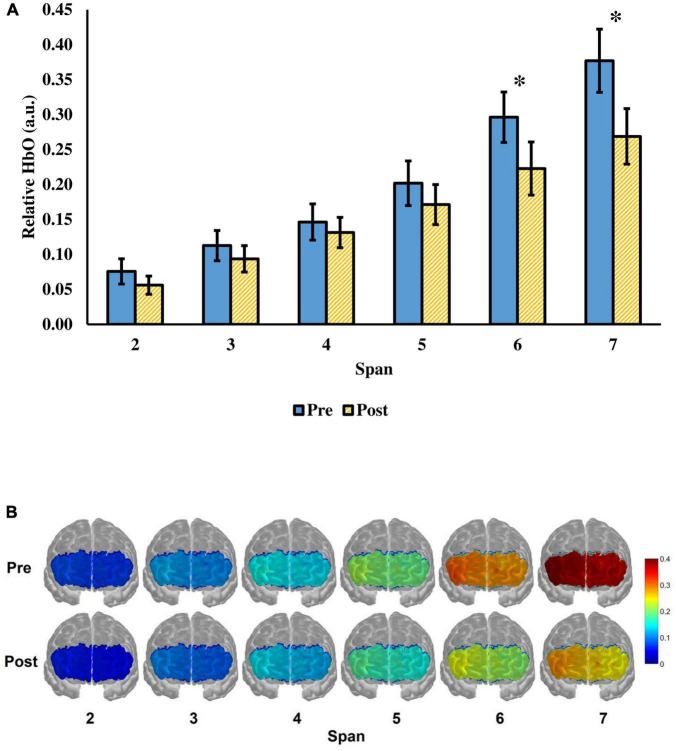
**(A)** The relative HbO of the visual span task from span 2 to 7 before and after a single tPBM session. The value of the relative HbO was averaged across the 16 fNIRS channels. Error bars represent one standard error of the mean. *p < 0.05. **(B)** Visualization of the relative HbO in the prefrontal cortex when the participants were performing the task. Red color represents greater prefrontal activation, whereas blue color represents lower prefrontal activation.

**FIGURE 4:**
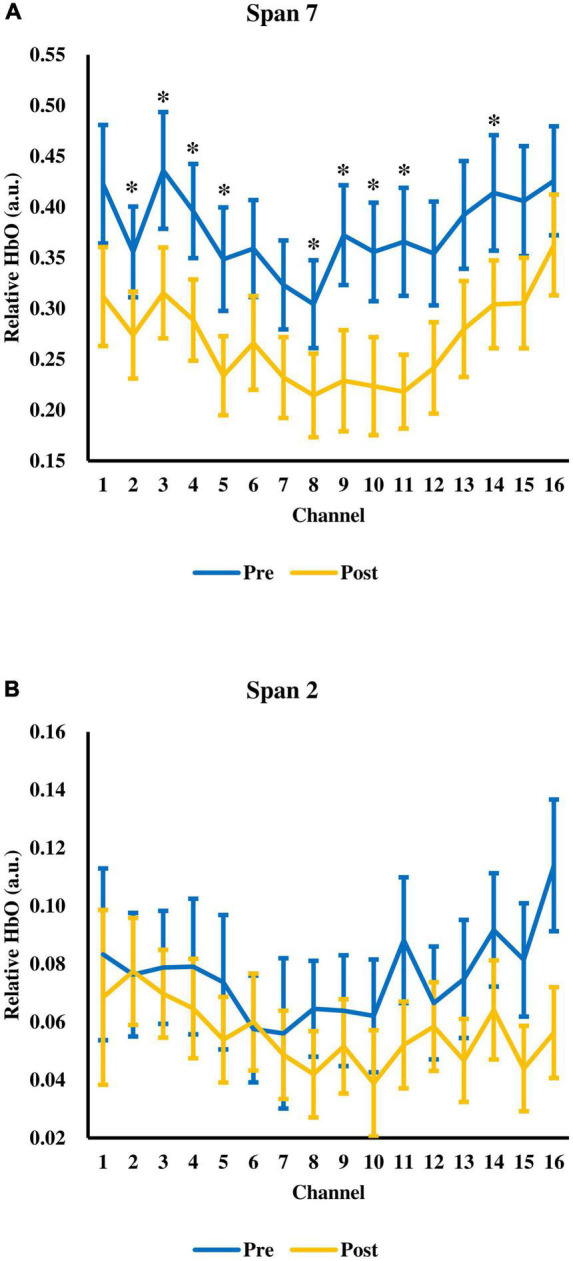
The relative HbO across 16 fNIRS measurement channels at **(A)** span 7 and **(B)** span 2. Error bars represent one standard error of the mean. *p < 0.05.

Next, the change in relative HbO after tPBM was evaluated. There was a significant time (pre-, post-assessment) × span (span 2 to 7) interaction, *F*(2.37,68.81) = 6.74, *p* = 0.001, $\eta_{p}^{2}$ = 0.19 (Figure 3). The main effect of time *F*(1.00,29.00) = 4.56, *p* = 0.041, $\eta_{p}^{2}$ = 0.14, and span, *F*(1.43,41.54) = 55.43, *p* < 0.001, $\eta_{p}^{2}$ = 0.66) were significant. There were significant decreases in relative HbO at span 6 and span 7 after tPBM. For span 6, the relative HbO decreased from 0.2962 (*SD* = 0.1973) to 0.2280 (*SD* = 0.2081), *t*(29) = 2.77, adjusted *p* = 0.029. For span 7, the relative HbO decreased from 0.3770 (*SD* = 0.2475) to 0.2688 (*SD* = 0.2175), *t*(29) = 3.07, adjusted *p* = 0.028. The changes were in medium effect size (span 6: *d* = 0.51; span 7: *d* = 0.56). While a significant decrease in relative HbO was observed across 9 channels in span 7, *t*(29) = 2.10–3.61, adjusted *p* = 0.018–0.0499, *d* = 0.38–0.66 (Figure 4A), none of the channels showed a significant decrease in relative HbO in span 2, *t*(29) = 2.70, adjusted *p* = 0.18–0.95 (Figure 4B).

### 3.2. Changes in task performance after tPBM

At pre-assessment, participants’ task performance varied from span 4 to 7, with an average of 5.30 blocks (*SD* = 0.92). Their performance (*M* = 5.63, *SD* = 1.00) was not statistically significant, *t*(29) = 1.62, *p* = 0.12, *d* = 0.30, after tPBM. However, it was found that the effects of tPBM varied among participants, with 12 participants (40.00%) having one or two more spans correct, 11 participants (36.67%) having unchanged performance, and 7 participants (23.33%) having one or two more spans incorrect. These three groups of participants did not differ significantly in terms of their changes in relative HbO at all the six spans *F*(2,27) = 0.10–0.89, *p* = 0.42–0.90, $\eta_{p}^{2}$ = 0.008–0.062, suggested that the change in the task performance was weakly related to the change in the relative HbO at each span.

### 3.3. Factors predicting the significant reduction of relative HbO after tPBM

**FIGURE 5:**
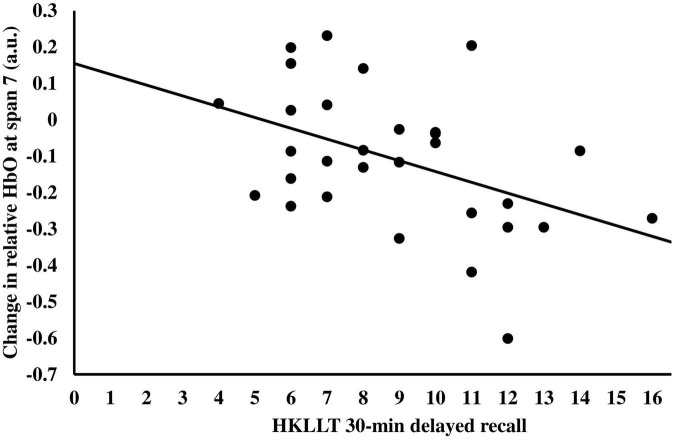
The relationship between the change in relative HbO at span 7 after tPBM and the 30-min delayed recall of HKLLT.

Given that the change of relative HbO at span 7 had a large variation (i.e., ranging from 0.2315 to −0.6009), correlation analysis was performed to investigate factors that may predict the change in relative HbO at span 7. It was found that the changes in relative HbO at span 7 were negatively correlated with the 30-min delayed recall score, *r* = −0.45, *p* = 0.013 (Figure 5), and the total learning score of the HKLLT, *r* = −0.38, *p* = 0.040, which suggested participants with better memory performance had more reduction in relative HbO in span 7 after tPBM. The demographic variables, including age and year of education, the other neuropsychological assessment scores, and the GAS and GDS scores, were not significantly related to the change of relative HbO at span 7, *r* = −0.27–0.19, *p* = 0.15–0.94. A stepwise linear regression was performed to determine predictors of the change of relative HbO at span 7 after tPBM. The abovementioned variables were entered into the prediction model. The result showed that only the 30-min delayed recall score could significantly predict the change of relative HbO at span 7 at post-assessment, β = −0.030, *p* = 0.013, with an *R*^2^ of 0.20, *F*(1,28) = 7.00, *p* = 0.013.

**FIGURE 6:**
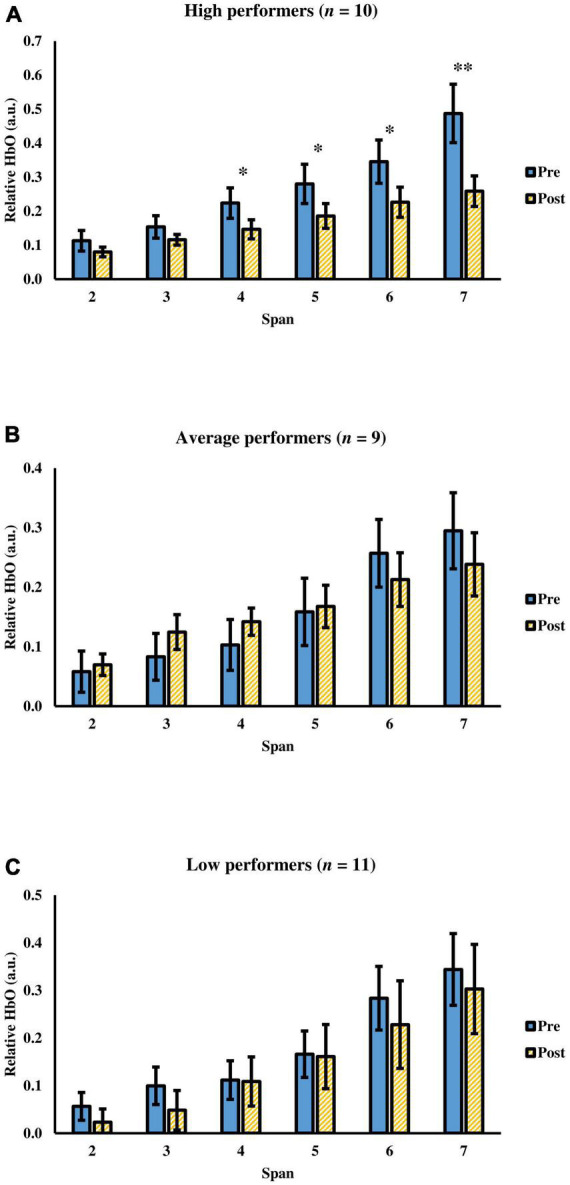
The relative HbO of the visual span task before and after a single tPBM session among **(A)** high performers, **(B)** average performers, and **(C)** low performers. Error bars represent one standard error of the mean. *p < 0.05 and **p < 0.01.

Since participants’ memory ability was negatively correlated with the change in cognitive efficiency after tPBM, they were further divided into three groups (i.e., high, average, and low memory performers) according to their *Z*-scores of the 30-min delayed recall in HKLLT: (1) high performers who scored at 67th percentile or above; (2) average performers who scored between 34th and 66th percentile; and (3) low performers who scored at 33rd percentile or below (Table 1). Mixed ANOVA showed significant group (three groups) × time (pre-, post-assessment) × span (span 2 to 7) interaction, *F*(5.06,68.29) = 2.54, *p* = 0.036, $\eta_{p}^{2}$ = 0.16. The time × span interaction was further evaluated in each group. The results showed that only the high performers showed significant interaction, *F*(1.62,14.60) = 7.71, *p* = 0.007, $\eta_{p}^{2}$ = 0.46, but not in average, *F*(1.82,14.58) = 3.46, *p* = 0.062, $\eta_{p}^{2}$ = 0.30, and low, *F*(5.00,6.00) = 1.76, *p* = 0.26, $\eta_{p}^{2}$ = 0.59, performers (Figure 6). For the high performers, there was a significant main effect of time, *F*(1.00,9.00) = 7.57, *p* = 0.022, $\eta_{p}^{2}$ = 0.46, and span, *F*(1.61,14.47) = 25.89, *p* < 0.001, $\eta_{p}^{2}$ = 0.74. The *Post hoc* comparison showed a significant decrease in relative HbO from span 4 to span 7 with medium to large effect size, *t*(9) = 2.37–3.31, *p* = 0.009–0.042, *d* = 0.75–1.05. Besides, a main effect of the span was found in terms of the change of relative HbO, *F*(1.62,14.60) = 7.71, *p* = 0.007, $\eta_{p}^{2}$ = 0.46, with more decrease of relative HbO at span 7 than other spans (mean difference = 0.11–0.20, *p* = 0.008–0.026). Besides, participants’ task performance was comparable after tPBM [from 5.56 to 5.70, *F*(2,27) = 0.046, *p* = 0.95]. There was no significant group × time interaction in terms of task performance, *F*(2,27) = 0.29, *p* = 0.75. In sum, it was found that tPBM seemed more beneficial to individuals with better memory ability.

**TABLE 1:** Comparison between low, average, and high performers.

Variables	Low (*n* = 11)		Average (*n* = 9)		High (*n* = 10)				
	*M*	*SD*	*M*	*SD*	*M*	*SD*	*F/χ^2^*	*P*	*Post hoc*
Age (year)	62.13	7.98	65.45	8.61	63.28	6.00	0.48	0.62	–
Gender (F/M)	6/5		3/6		8/2		4.23	0.12	–
Education (year)	12.27	2.65	10.33	3.97	12.80	3.08	1.52	0.24	–
Geriatric Anxiety Scale	4.91	3.70	5.89	4.40	9.50	5.42	2.90	0.07	–
Geriatric Depression Scale	3.09	2.43	3.44	2.40	5.60	3.24	2.53	0.10	–
**Rey-O**									
Copy	30.41	3.90	30.06	3.58	31.45	3.24	0.39	0.68	–
Immediate recall	14.27	5.16	12.67	3.58	15.35	7.63	0.52	0.60	–
Delayed recall	13.36	4.00	13.06	3.75	15.00	6.98	0.41	0.67	–
**HKLLT**									
Total learning	22.00	5.57	22.89	3.98	29.70	5.36	6.98	0.004	High > Average, Low
10-min delay	7.09	1.81	8.89	2.15	12.00	2.54	13.53	<0.001	High > Average, Low
30-min delay	6.09	1.04	8.56	1.13	12.20	1.75	54.50	<0.001	High > Average > Low
**Digit Span**									
Digit Span Forward	7.45	1.51	7.33	1.58	7.60	1.17	0.083	0.92	–
Digit Span Backward	5.00	1.55	5.00	0.71	5.50	1.08	0.58	0.56	–
Category Fluency Test	32.91	11.18	30.56	7.65	34.30	9.94	0.35	0.71	–
Visual span task	5.09	0.83	5.33	1.32	5.50	0.53	0.51	0.60	–
**Relative HbO of the visual span task (a.u.)**									
Span 2	0.0564	0.0966	0.0580	0.1039	0.1130	0.0959	1.07	0.36	–
Span 3	0.0996	0.1305	0.0830	0.1178	0.1537	0.1044	0.95	0.40	–
Span 4	0.1116	0.1344	0.1030	0.1282	0.2237	0.1416	2.47	0.10	–
Span 5	0.1659	0.1618	0.1585	0.1697	0.2802	0.1824	1.58	0.22	–
Span 6	0.2835	0.2216	0.2569	0.1706	0.3456	0.2012	0.50	0.61	–
Span 7	0.3440	0.2499	0.2948	0.1917	0.4874	0.2716	1.66	0.21	–

There were 11 participants in the low performer group because the 11th lowest participant has the same score as the 10th lowest participant. HKLLT, Hong Kong List Learning Test; Rey-O, Rey–Osterrieth Complex Figure Test.

## 4. Discussion

Based upon the findings of previous studies that demonstrated improved cognitive functions such as memory (Barrett and Gonzalez-Lima, 2013; Hwang et al., 2016), attention (Barrett and Gonzalez-Lima, 2013; Jahan et al., 2019), and executive function (Blanco et al., 2015; Chan et al., 2019a), the present study aimed to investigate the underlying mechanism of this improvement. fNIRS was adopted to measure the change in brain concentration of HbO in cerebral blood flow when the participants were performing the visual span task. Previous studies demonstrated that HbO is a reliable indicator of mental effort used in completing a cognitive task (Causse et al., 2017; Csipo et al., 2021). That is, the more difficult the task, the higher the HbO level. The present study showed that participants showed significantly lower relative HbO at a longer span (i.e., span 7) after tPBM, and their behavioral performance was comparable to the baseline. In other words, after tPBM, participants used less effort in the difficult trials and maintained their performance level. Therefore, the present findings provided evidence that tPBM may enhance cognitive efficiency by reducing the amount of mental effort necessary for completing cognitive tasks at a difficult level. Several beneficial effects were found during tPBM, including an increase in adenosine triphosphate levels and an increase in the cerebral concentration of HbO (de Freitas and Hamblin, 2016; Tian et al., 2016). These effects lead to enhanced brain metabolic activity, which may reduce the HbO level, indicating less effortfulness in the task.

The reduced HbO after tPBM is consistent with previous studies on healthy younger adults (Chan et al., 2021a) and adults with MCI (Chan et al., 2021b), which also reported improved cognitive efficiency after a single trial of tPBM was given. In comparison to previous transcranial pulsed laser stimulation (tPBM) studies that reported a beneficial effect on cognitive performance in younger adults (e.g., Barrett and Gonzalez-Lima, 2013; Hwang et al., 2016; Jahan et al., 2019), the present study found only an effect on cognitive efficiency. This may be attributed to the relatively brief dosage of light and the different sample populations employed, as the present study focused on older adults. Still, the present study, together with our previous study on younger adults, suggests that tPBM may enhance cognitive efficiency in both young and older adults. In the previous study on younger adults, a verbal working memory task (i.e., the n-back task) was utilized, whereas a visual working memory task (i.e., the visual span task) was employed in the present study on older adults. These findings suggest that the observed improvement in cognitive efficiency after tPBM was not limited to a single type of working memory processing.

The present finding suggested that older adults with better memory may benefit more from the tPBM. That is, the correlation and regression results found that the change in mental effort after tPBM, as measured by the change in relative HbO at span 7, was negatively associated with the score of the HKLLT 30-min delayed recall at pre-assessment. In addition, after dividing the participants into high, average, and low memory performers, the results found that only the high performer group showed significantly reduced relative HbO from span 4 to span 7 after tPBM. With similar performance as in the pre-assessment, the present study suggested that older adults with better memory seem to benefit more from tPBM in enhancing cognitive efficiency. In other words, the level of memory ability may be a predictor of the effectiveness of tPBM on individual subjects. Previous studies found that the size and activity of the hippocampus were associated with better memory in older adults (see Bettio et al., 2017 for review). This may explain why older adults with better memory could respond better to tPBM. However, it is noted that the present study did not measure the size and activity of the hippocampus. Future studies are needed to test this hypothesis. Besides, the non-significant correlation between depression and anxiety symptoms suggested that psychological status may not be a predictive factor in the effectiveness of tPBM.

The fNIRS device utilized a similar wavelength of light as the tPBM device, which may have elicited a cognitive enhancement effect akin to tPBM. However, regardless of whether the tPBM effect was mainly due to the fNIRS device or the tPBM device, it did not affect the conclusion of the present study, which suggests that tPBM may enhance cognitive efficiency in older adults. Additionally, in our previous study, we calculated that the dosage delivered by the fNIRS device was about 0.2 J/cm^2^ for both wavelengths (Chan et al., 2021a), which was nearly negligible compared to the tPBM device used in the present study (i.e., 7 J/cm^2^). Therefore, it is unlikely that the fNIRS device induced a significant cognitive enhancement effect in older adults, particularly those with mild cognitive impairment, as our prior study found no significant improvement after sham stimulation (Chan et al., 2021a).

Since the participants had to perform the visual span task twice, the reduction in HbO observed during post-assessment may be due to increased familiarity with the task, resulting in a reduced cognitive load rather than an effect of tPBM *per se*. To the best of our knowledge, there was no previous study that examined changes in HbO over multiple iterations of the same visual span task employed in the present study on healthy older adults. Instead, we examined the effect of tPBM on MCI using a sham tPBM device and found that neither task performance nor HbO changed significantly (Chan et al., 2021a). Future studies may consider including a sham-treated control group when examining the effect of tPBM on healthy older adults to better account for potential confounding factors, such as task familiarity.

Although the present study suggested that individuals with better memory may benefit more from tPBM, it does not imply tPBM is ineffective for those with memory problems, such as amnestic MCI and dementia. It is noted that only a single tPBM session was given to the participants. With less amount of energy delivered, the beneficial effect of tPBM may not be observed in individuals with relatively lower memory performance at baseline. From the existing tPBM studies on MCI and dementia, multiple treatment trials with higher total doses were commonly applied (e.g., Saltmarche et al., 2017; Chao, 2019; Salehpour et al., 2019b; Chan et al., 2021c). More importantly, positive effects were also reported after tPBM, including improved general cognitive function (Saltmarche et al., 2017; Chao, 2019; Salehpour et al., 2019b), memory (Chan et al., 2021c), better sleep and less anxiety and wandering (Saltmarche et al., 2017), and reversal of olfactory dysfunction (Salehpour et al., 2019b). Given the positive effect of tPBM (Chan et al., 2019b; Lee et al., 2022), tPBM has the potential to be developed as a cognitive intervention in the future.

## 5. Conclusion

In conclusion, the present study demonstrated that tPBM significantly reduces mental efforts in completing a cognitive task with a higher difficulty level in older adults, thus enhancing cognitive efficiency. Given that cognitive inefficiency was found to be related to a high risk of future cognitive impairment (Pettigrew and Soldan, 2019), tPBM may have the potential to be served as an effective intervention that prevents abnormal cognitive deterioration.

## Data availability statement

The raw data supporting the conclusions of this article will be made available by the authors, without undue reservation.

## Ethics statement

The studies involving human participants were reviewed and approved by the Joint Chinese University of Hong Kong–New Territories East Cluster Clinical Research Ethics Committee. The patients/participants provided their written informed consent to participate in this study.

## Author contributions

T-lL: conceptualization, methodology, formal analysis, investigation, writing—original draft, and visualization. AC: conceptualization, methodology, resources, writing—review and editing, and supervision. Both authors contributed to the article and approved the submitted version.
